# Women’s empowerment as a driver to household food security in the Eastern Cape Province, South Africa: a structural equation model approach

**DOI:** 10.3389/fsoc.2026.1758912

**Published:** 2026-03-23

**Authors:** Chenaimoyo Lufutuko Faith Katiyatiya, Thobeka Ncanywa

**Affiliations:** 1 Faculty of Education, Walter Sisulu University, Mthatha, South Africa; 2 Directorate of Research and Innovation, Walter Sisulu University, Mthatha, South Africa

**Keywords:** agriculture, food security, rural, South Africa, structural equation model, women’s empowerment

## Abstract

Livelihood resilience and food security remain central in achieving sustainable development. The study investigated the structural relationships between women’s empowerment, access to resources, agricultural participation and food security in urban and rural households in the Eastern Cape Province, South Africa. The study employed the Structural Equation Modelling (SEM) to analyze data from 19,017 participants utilizing the 2023 and 2024 South Africa General Household Surveys to assess the direct and indirect effects of socio-economic and infrastructural factors on livelihood outcomes. The results reveal that women’s empowerment has an indirect effect reducing food insecurity through enhanced access resources (*p* ≤ 0.001). Urban residence and improved sanitation significantly influenced resource accessibility (*p* ≤ 0.001). Larger household size constrains resource access, highlighting intra-household vulnerability (*p* ≤ 0.001). Agricultural participation is strongly influenced by urban–rural location and household size (*p* ≤ 0.001). Although the SEM showed negative association of agricultural participation and food insecurity, the logistic regression highlighted a positive influence (*p* ≤ 0.001). The findings also indicate that resource access, empowerment, agricultural participation, household size and urban residence have direct effects on food insecurity (*p* ≤ 0.001). Empowerment has strong direct effects on resource access. Resource access and empowerment have direct effects on agricultural participation (*p* ≤ 0.001). Agricultural participation, access to resources and food insecurity had direct effects on agriculture sales (*p* ≤ 0.001). Empowerment had indirect effect on food security through improved resource access and reduced agricultural participation (*p* ≤ 0.001). Resource access had the strongest mediating factor. Empowerment and income access exert negative but significant effects, indicating potential opportunity costs between off-farm income and agricultural engagement. Rural households have higher risks of food insecurity than in urban areas. It was concluded that women’s empowerment and access to improved basic services promote income opportunities that reduce food insecurity and support agricultural commercialization. The study highlights that, targeted interventions to improve resilience and nutrition among households is imperative in supporting sustainable development goals related to gender equality and food security.

## 1 Introduction

Food security is significant in sustainable development especially in developing regions where agriculture sustains rural livelihoods and urban food supply (Mazenda et al., 2025; Gunapala et al., 2025). The contribution of women in agriculture and food systems is fundamental in achieving food security among households (Masuku et al., 2023; Rudolph et al., 2024). Globally, women play a central role in agriculture as they are known to drive household agricultural activities for household survival and improving livelihoods (Von Maltitz and Bahta, 2024; Hidrobo et al., 2024). In South Africa, research has shown that, women are the backbone of food production and management (Nkwana and Mazenda, 2025; Mazenda and Nkwana, 2025).

Despite their contribution, women are often faced by significant challenges, limiting their effectiveness in agricultural production and household food security (Von Maltitz and Bahta, 2024; Maltitz and Bahta, 2021). These challenges include limited access to land, credit, technology, extension services and decision making comparable to men (Ashagidigbi et al., 2022; Mkuna and Wale, 2023). This gender gap in agricultural empowerment suppresses food security and productivity of the agriculture sector (Adebayo and Worth, 2024). However, narrowing these gaps is associated with higher yields, better child nutrition and greater resilience to shocks. Closing gender gaps in agriculture through equitable access to resources enhances resilience, productivity and nutrition (Bryan et al., 2024). Gender equity improves agrifood systems’ shock resilience by utilizing women’s expertise in adaptation practices (Njuki et al., 2022). Equal resource access increases women’s agricultural yields by 20–30%, contributing to 2.5–4% rise in national output. Women’s control over resources results in diverse, nutrient-rich crops and better household health spending, reducing global malnutrition rates by 12–17% and improving child health outcomes (Harris-Fry et al., 2025; Gender Climate Change and Nutrition Integration Initiative (GCAN), 2022).

Globally, women’s empowerment and food security have gained attention as evidenced by the Sustainable Development Goals (SDG), particularly SDG 2 on zero hunger and SDG 5 on gender equality (Lufuke et al., 2023). Research has shown that women smallholder farmers face restricted access to credit, inputs, land and information, limiting productivity and climate adaptation; financial constraints and input shortages top barriers (Mwalupaso et al., 2025). Mwalupaso et al. (2025) also reported that intensifying shocks exacerbated women’s burden with pests, diseases and environmental stressors affecting them harder due to poverty and marginal lands reported structural inequalities such as social norms, low youth engagement ad inadequate training hinder sustainability. Despite the challenges, emerging opportunities exist for women in agriculture. These include inclusive climate-smart agriculture adoption that is gender sensitive, female extension workers and land reform boosts resilience in Sub-Saharan Africa. Information and communication technologies, and tailored finance empower decision-making, yields and adaptation for women. Integrated solutions such as mechanization, youth programs and farmer groups address generational renewal and equity (Awoke et al., 2025).

It is, therefore, critical to conduct localized research examining how empowerment dimensions intersect with food security across urban and rural areas. Empowering women is essential for improving household food security and agricultural sustainability (Von Maltitz and Bahta, 2024), yet discrepancies exist between urban and rural women’s empowerment and its effects on these outcomes. Despite investments in agricultural development, food insecurity persists in developing countries, suggesting that agricultural participation alone is in sufficient. This paper examines these dynamics, highlights gender disparities and advocates gender-inclusive agricultural policies. It particularly focuses on gender disparities in South Africa, highlighting vulnerabilities in female-headed households especially in urban areas, exacerbated by structural barriers and economic challenges. The study addresses gaps in understanding women’s empowerment and seeks to provide actionable insights for policymakers to achieve goals related to food security and gender quality. The study utilizes the Structural Equation Modelling (SEM) to provide an understanding of complex relationships between women’s empowerment, access to services, agricultural participation and food security. The SEM is used to test the causal pathways, reveal direct and indirect effect and clarifying mechanisms that single-equation models overlook, thereby enhancing understanding of how access constraints influence livelihood outcomes.

Therefore, the study addresses the following research questions:

1. What are the direct and indirect relationships between women’s empowerment and household food security?
2. Do these relationships differ between rural and urban areas?

The Eastern Cape is one of the marginalized provinces in South Africa. It is characterized by significant rural inequalities and high unemployment, with food security remaining a persistent challenge (Nontu et al., 2024). Reports using the Food Insecurity Experience Scale highlight patterns of food insecurity from 2019 to 2023, showing significant urban–rural disparities and the importance of women’s roles within households (Statistics South Africa, 2025). While the General Household Survey indicates some improvements in sanitation and other services affecting food access, disparities persist, impacting nutrition and food safety. Emerging literature highlights the challenges and opportunities for women and smallholder farmers in agriculture, where declining farming engagement contrasts with the potential for market access driven by irrigation and organization. This study, therefore, utilized the structural approach to investigate the relationship between women’s empowerment, access to resources, agricultural participation and food security in urban and rural households in the Eastern Cape Province, South Africa.

## 2 Literature review

### 2.1 Empirical literature

The study adopts the Kabeer’s Empowerment Framework (Kabeer, 1999). The framework defines empowerment as the process involving resources, decision making and achievements such as food security (Kabeer, 1999). The framework is widely applied in development to explain how women’s control over resources and decisions leads to improved food security (Kabeer, 1999, 2005). Guided by the Kabeer’s empowerment theory, this study defines women’s empowerment as a multidimensional process involving resources, agency and achievements. Resources such as land, credit and education enable or hinder women’s actions. Agency signifies women’s ability to make strategic choices related to household production and food decisions (Kunzekweguta et al., 2025). Achievements are the results of exercising that agency specifically regarding household food security in availability, access, utilization and stability. The framework posits that greater access to resources enhances women’s agency leading to decisions that improve food security. Empowerment impacts household food security both directly and indirectly through improved livelihoods and equitable resource distribution (Kunzekweguta et al., 2025).

Empowered women tend to prioritize income towards food, healthcare and education. Masuku et al. (2023) found that, in South Africa, women’s access to land and agricultural credit is hindered by customary systems and institutional barriers. Empowerment initiatives such as cooperatives and training improve food availability and dietary diversity. Key elements in women’s empowerment in agriculture include access to resources, asset ownership, decision making power and participation in household matters (Mazenda and Nkwana, 2025). This enhances nutrition and reduce food insecurity through stronger agency supported by education and policies.

#### 2.1.1 Global evidence (Sub-Saharan Africa, South Asia and East Africa)

In Sub-Saharan Africa, gender inequality affects agricultural productivity and food security (Daidone et al., 2020). Previous studies have shown the women’s role in resource allocation and production decisions enhances household food security and nutrition. Evidence from different countries shows that reducing gender disparities in access to land, inputs and finance leads to increased farm productivity and improved food security, while persistent inequalities hinder output and resilience (Jemaneh and Shibeshi, 2023; Egah et al., 2023; Akalu and Wang, 2023; Elum and Emodi, 2025; Sarker et al., 2024; Sibiya and Oluwatayo, 2023). According to Alkire et al. (2013), the Women’s Empowerment in Agriculture Index (WEAI) highlights decision-making power, income control and leadership crucial factors related to better production and welfare results.

#### 2.1.2 South African context

Statistics South Africa (2023a) revealed that female-headed households are more prone to moderate or severe food insecurity, emphasizing the link between empowerment and food security in South Africa. Ngarava et al. (2019) reported that in the Eastern Cape, urban women typically engage in informal markets, backyard gardens and microenterprises for their income and food. Rural women in the province, rely on agriculture and livestock, influencing empowerment and food security levels (Ngarava et al., 2019). Urbanization presents economic participation opportunities for women, but rural areas face more significant structural barriers. Women’s empowerment is essential for enhancing agricultural productivity and resilience to challenges like climate change (Nontu et al., 2024). Nevertheless, inequalities in resource access between urban and rural women emphasize the necessity for targeted interventions.

Nontu et al. (2024) indicate that women’s empowerment in home and community gardening is crucial for food availability and diversity, particularly where formal jobs are scarce. However, these initiatives rely on access to resources such as water and support services. Urban agriculture is developing as a livelihood strategy, led by women who facilitate cultivation and marketing enhancing food diversity and resilience to price fluctuations (Khumalo and Zenda, 2025; Mkhize et al., 2023). Nonetheless, challenges such as insecure land tenure and limited access to water hinder scalability.

The WEAI framework has become a central tool in measuring women’s agricultural empowerment globally. Thobejane et al. (2023) and Malapit et al. (2019) show that empowerment domains, production decisions, income control, leadership, asset ownership, and time strongly correlate with productivity and food security outcomes. In the South African context, programs targeting these domains can enhance both agricultural performance and food security. Huyer et al. (2024) highlights that gender productivity gaps narrow when women access comparable resources, but widen during periods of climate stress. Women’s budgeting and production roles are critical for buffering households from price and input shocks, particularly in rural provinces such as the Eastern Cape (Nontu et al., 2024; Mthethwa and Wale, 2023).

#### 2.1.3 Urban–rural comparative perspectives

Empirical literature identifies three testable pathways in the Eastern Cape: (a) women’s empowerment and decision-making reduce food insecurity probabilities (Food Insecurity Experience Scale/Household Food Insecurity Access Scale measures); (b) women-led agriculture improves household food availability and diet diversity; and (c) access to services such as water, sanitation and energy enhances agricultural stability and food utilization. These findings indicate the importance of integrating gender-sensitive interventions into agricultural and food security programs in the province (Statistics South Africa, 2025; FAO et al., 2023).

#### 2.1.4 Critiques and debates on women’s empowerment

Women’s empowerment strategies in agriculture are criticized for causing unintended effects such as increased time poverty and added labor burden (Quisumbing et al., 2021). Programs such as cash crops and hybrid adoption may intensify women’s workloads, affecting their domestic responsibilities and welfare. Barriers such as male-dominated services, restrictive social norms, and competition from men can further disempower women. Despite the promotion of women’s empowerment as beneficial globally, research argue that economic participation does not equate to control over resources or decision making. Consequently, emerging challenges ranging from intra-household tension and exposure to gender-based conflict.

Debates on women’s empowerment explore whether it should be viewed as an individual or household level construct. The WEAI measures both individual agency and household parity (Costa et al., 2023). Research indicates that women’s control over assets can enhance their bargaining power, influencing resource allocation within households (Connors et al., 2023; Quisumbing et al., 2023). However, empowerment outcomes vary by context. Household wealth or entrepreneurship do not consistently translate to individual empowerment (Quisumbing et al., 2025). Individual approach emphasize women’s resources and agency, consistent with Kabeer’s framework, while critics highlight the need to consider relational power dynamics in smallholder farming (Costa et al., 2023; Quisumbing et al., 2025). Household-level perspectives focus on bargaining, decision making processes and the balance between individual autonomy and collective welfare, proposing that true empowerment involves altering intra-household relations for collective well-being (Costa et al., 2023; Quisumbing et al., 2023).

Empowerment can negatively impact household food security when women’s income-generating activities require time that could otherwise be spent on food production. Men’s control over resources may decrease crop yields and variety, and women’s increased empowerment could lead men to reduce their contributions to food budgets (Murugani and Thamaga-Chitja, 2019). Therefore, nutrition-sensitive agriculture could exacerbate women’s existing burdens, potentially harming household nutrition outcomes. It is essential to recognize that women’s empowerment does not always correlate with improved food security; factors such as increased income-generating activities can reduce time for food related tasks and control over income does not guarantee prioritization of food expenses (Quisumbing et al., 2021). These complex dynamics highlight non-linear relationship between empowerment and food security, particularly in resource-limited and climate-vulnerable environments.

### 2.2 Conceptual framework

**Figure 1:**
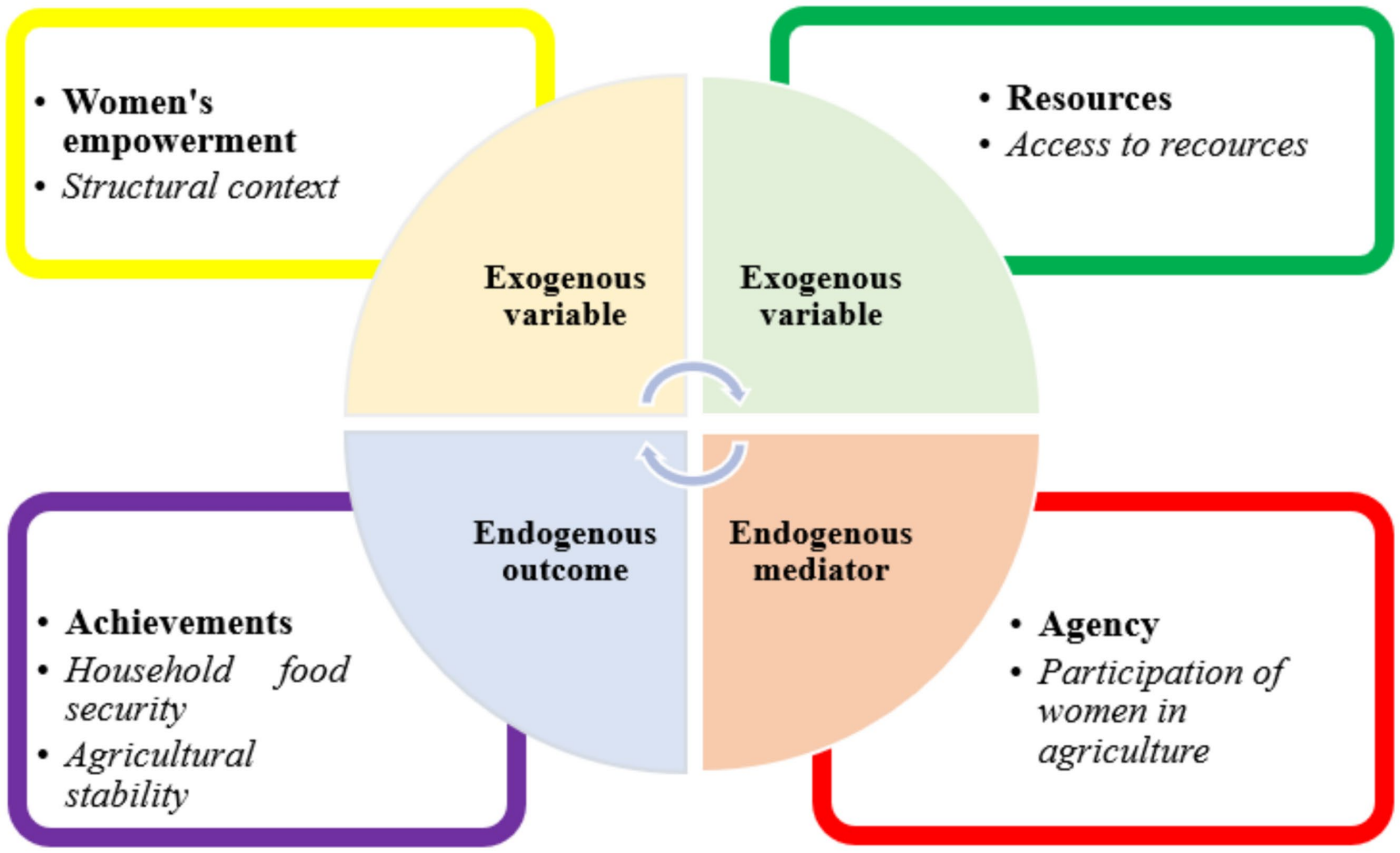
Conceptual framework illustrating pathways from women’s empowerment to household food security in Eastern Cape Province, South Africa. Boxes represent constructs; arrows indicate hypothesized relationships. Adapted from Kabeer’s Empowerment framework (Kabeer, 1999, 2005).

Based on the literature review, a conceptual framework employed in the study is presented on Figure 1. Figure 1 illustrates how women’s empowerment and access to productive resources contribute to food security and agricultural stability across urban and rural households in the Eastern Cape, South Africa. The framework highlights women’s active involvement as a central driver influencing key outcomes by increasing access to productive resources, strengthening agency with household and community agricultural practices and promoting adaptive strategies to climate and economic shocks (Tantoh et al., 2026). This paper contributes to the literature by empirically examining the linkages of women’s empowerment in agriculture and food security outcomes in the Eastern Cape. It establishes that women’s participation enhances resource utilization, diversifies income recourses and improves nutritional status resulting in improved household food security (Haque et al., 2024). It also identifies feedback loops where increased agricultural resilience supports sustained women’s engagement and decision-making. The conceptual framework provides guidance for research design and suggests mechanisms through which gender-inclusive interventions can transform food security and resilience. This will serve as a theoretical lens and roadmap for policy recommendations.

## 3 Materials and methods

### 3.1 Data sampling

This study utilized cross-sectional data from the 2023 and 2024 General Household Surveys (GHS) for households in the Eastern Cape, South Africa. The GHS is a national representative survey that collects information on socio-demographics, household food security, agricultural practices, and access to basic services. The data provides information on household demographics, income sources, labor market participation, education, access to service, and food security outcomes. The data is collected at household level, capturing aspects such as income, food access, housing conditions, and at individual level, focusing on education, employment, gender and age. This allows for the construction of gender-disaggregated indicators. The dataset contains information on food access, coping behaviour, hunger, female headship, women’s education, employment as well as income participation. The GHS employed a design with proportional stratification. The first stage involved selecting primary sampling units based on provincial and population characteristics, followed by systematic sampling of dwelling units, Trained Statistics South Africa (StatsSA) survey officers administered the questionnaires across all nine provinces of South Africa. For the purpose of this study, analyses were restricted to households residing in the Eastern Cape province, one of the marginal provinces in South Africa. The total data sample at national level for the two years is 141,614. Response rate for Eastern Cape households was 95.6 and 94.1% in 2023 and 2024, respectively (Statistics South Africa, 2023a, 2024). However, for this study, a total of 19,017 individuals were considered for the Eastern Cape Province, out of 141,614 individuals at national level. Survey weights and primary sampling units were applied to account for the complex survey design. The current study analyzed data related to socio-economic characteristics, access to services and food security related issues.

### 3.2 Empirical analysis

Descriptive and bivariate analyses were performed to summarize household characteristics and by residence food insecurity status, with differences tested using survey-adjusted chi-square tests for categorical variables and *t*-tests for continuous variables. The primary outcome, household food insecurity, was derived from survey items assessing insufficient food for adults and children and concern about food availability. Food insecurity variable was drawn from the Food Insecurity Experience Scale (FIES) developed by Food and Agriculture Organization and has an 8-item survey based metric. Households reporting any of these conditions were coded as food insecure (1), otherwise as food secure (0). The variables used are shown in Table 1.

**Table 1:** Description of variables.

Variable name	Description	Type
Food Insecurity Experience Score_Multidimensional Severity Score (FIES_MSS)	Household food insecurity	Binary
Female head	Sex of household head	Binary
Women empowerment index	Share of employed adult women in household	Continuous
Agriculture participation	Household participates in agricultural activities	Binary
Household size	Total number of individuals in a household	Continuous
Women education average	Household average years of education (adult women ≥15 years)	Continuous
Grants (any)	Household receives any grants	Binary
Remittance (any)	Household receives remittances	Binary
Improved water	Improved drinking water source	Binary
Improved sanitation	Improved sanitation facility	Binary
Clean cooking energy	Clean cooking energy used	Binary
Household asset	Household asset ownership	Binary
Urban	Geographic location (urban, rural)	Binary

Household food insecurity, was modelled using a survey-weighted logistic regression


$$\begin{array}{l} \text{logit}(P(\text{FIES}\_{\mathrm{MSS}}_{i}=1))\\ =\beta_{0}+\beta_{1}\text{female}\_{\text{head}}_{i}+\beta_{2}\text{women}\_\mathrm{emp}\_{\text{index}}_{i}\\ +\beta_{3}\mathrm{agr}\_{\text{participate}}_{i}+\beta_{4}{\left(\text{female}\_\text{head}\times \mathrm{agr}\_\text{participate}\right)}_{i}\\ +\gamma^{\prime }X_{i}+\delta_{1}{\text{urban}}_{i}+\delta_{2}{\text{year}}_{t}+\varepsilon_{i}. \end{array}$$ (1)

Where $\text{FIES}\_{\mathrm{MSS}}_{i}$ indicates household food insecurity (Dependent variable is variable with 1 representing households *i* experiences moderate or severe food insecurity, while 0 represents otherwise).

*β*_0_ is the intercept which represents the baseline log-odds of food insecurity for the reference household.

*β_1_* female_head*i* presents a binary variable equal to 1 if the household is female-headed. *β_1_* captures the gender gap in food insecurity among non-agricultural household. A positive coefficient suggests female-headed households are more food insecure while a negative coefficient suggests the opposite.

*β_2_* represents a composite index which captures women’s empowerment such as decision-making, access to resources. *β_2_* < 0 implies that higher women’s empowerment reduces the likelihood of moderate or severe food security. This coefficient tests whether empowerment operates as a protective factor against food insecurity.

*β_3_* indicates whether the household participates in agricultural activities. *β_3_* measures the effect of agricultural participation for male-headed households. A negative coefficient implies agriculture improves food security through own production, income or livelihood diversification.

*β_4_* is an interaction effect which captures whether the effect of agricultural participation on food insecurity differs between female- and male-headed household. The total effect of agriculture for female-headed households is: *β_3_* + *β_4_*

$\gamma^{\prime }X_{i}$ represents household-level covariates,

*δ*_1_ urban*_i_* represents the structural differences between urban and rural households

*δ*_2_ year*_t_* represents year fixed effects, and

$\varepsilon_{i}$ is the error term.

The dependent variable was household food insecurity, derived from survey variables assessing insufficient food for adults and children, as well as worry about food availability. Household-level variables were derived from information provided by household members aged 15 years and older. Females heading a household were coded based on the sex of the household head. Women’s education was calculated as the average years of formal schooling among adult women in the household. Women’s empowerment rate reflects the proportion of adult women within households who were employed.

Agricultural participation refers to whether any adult household member is engaged in agricultural activities. An interaction term between female household heads and agricultural participation was included to assess whether the effect of agricultural participation differs in female-headed households.

Other covariates were measured at the household level and included: household size, access to basic services (improved water, improved sanitation, clean cooking energy), household assets (standardized asset index), and income sources (receipt of grants and remittances). Urban residence and survey year were included to account for temporal and spatial heterogeneity. Disaggregated analyses were conducted for rural and urban households to examine heterogeneity by area of residence.

The hypotheses tested were that women-headed households experience significantly different food insecurity compared to male-headed households. Higher levels of women’s empowerment are associated with a lower probability of food insecurity. Agricultural participation reduces food insecurity among male-headed households. The food security impact of agricultural participation differs between female and male headed households.

In addition to the logistic regression analysis, structural equation modelling (SEM) was employed to examine the interrelationships between women’s empowerment, agricultural participation, and household food insecurity within a unified analytical framework. The SEM allows for the simultaneous estimation of multiple relationships and is particularly useful for assessing indirect pathways and latent constructs.

Women’s empowerment was measured using a composite index constructed through principal component analysis (PCA). The index was derived from three indicators: women’s employment rate, average years of formal education among adult women in the household, and female headship. Agricultural participation and household food insecurity were specified as observed variables. The SEM framework was used to assess both the direct effects of women’s empowerment and agricultural participation on food insecurity, and the indirect pathways linking women’s empowerment to food security through agricultural engagement.

Given the presence of categorical variables and departures from normality, the SEM estimation relied on methods robust to non-normality. Model adequacy was evaluated using standard SEM fit relative magnitude of associations rather than strict causal inference. All the data were analyzed using STATA 18.

## 4 Results

### 4.1 Descriptive analysis

Table 2 presents descriptive statistics for the sample of 19,017 households from the Eastern Cape included in the analysis. On average, 35% of households were classified as food insecure. The proportion is high representing a structurally disadvantages proportion the population, although Eastern Cape is known historically to be vulnerable to food insecurity. The proportion highlights the analytical relevance of examining empowerment, agricultural participation and resource access as potential pathways influencing household food security outcomes. Slightly more than half of the households (54%) were headed by women participants, and approximately 47% participated in some form of agricultural activities. The mean female employment rate across households was 26%, and the average household consisted of 5 members. The employment rate lower possible due to the age description for adults (≥15) used in the study which could have included school-going females. Adult women reported an average of 9 years of formal education.

**Table 2:** Descriptive statistics (*N* = 19,017).

Variable	Mean	% of sample	Standard deviation	[95% Conf. interval]
Food insecurity experience scale	0.351	35.1	0.477	(0.343, 0.358)
Female head	0.568	56.8	0.499	(56.03, 57.57)
Women employment index	0.468		0.446	(0.460, 0.475)
Agriculture participation	0.465	42.7	0.499	(0.457, 0.472)
Household size	5.236		2.959	(5.201, 5.283)
Women education average	9.266		2.958	(9.509, 9.598)
Grants	0.821	82.1	0.384	(0.806, 0.819)
Remittance	0.166	16.6	0.372	(0.166, 0.178)
Improved water	0.726	72.6	0.454	(0.719, 0.732)
Improved sanitation	0.874	87.4	0.332	(0.873, 0.883)
Clean cooking energy	0.816	81.6	0.387	(0.829, 0.839)
Standardized household asset index	−0.292		0.946	(−0.263, −0.232)
Urban	0.447	44.7	0.497	(0.489, 0.505)

Regarding household income and support, 82% of households reported receiving social grants, while 17% reported receiving remittances. Access to improved basic services was relatively high, with 71% of households having access to improved water sources, 87% to improved sanitation facilities, and 82% using clean cooking energy. The standardized household asset index had a mean of −0.29, reflecting variability in wealth and resources across the sample. Generally, the mean of −0.29 implies that households in the Eastern Cape own fewer assets on average in comparison. This does reflect structural poverty and deprivation which is a characteristic of the Eastern Cape province as compared to other provinces. A standard deviation of 0.946 indicates substantial variation in asset ownership, suggesting that some households are extremely poor when it comes to asset ownership as compared to others. Finally, approximately 45% of sample households were located in urban areas.

### 4.2 Food insecurity status and residential characteristics

Table 3 compares household characteristics by food security status. Female-headed households were more likely to be food secure (61.58%), while food-insecure households had lower female employment rate. Larger household size (5 members), and lower women’s education (8.89%) were also associated with higher food insecurity. Households that were food secure tended to have greater access to social support, improved water and sanitation, clean cooking energy, and higher household assets. All the observed differences were statistically significant (*p* ≤ 001), highlighting key socio-demographic and household factors associated with food insecurity in the Eastern Cape.

**Table 3:** Household characteristics by food security status.

Variable	Food security (%)	Food insecurity (%)	Chi-square/*t*-statistics
Female-headed	61.58	38.42	105.33***
Women empowerment index	0.13	0.07	−10.85***
Agriculture participation	64.71	35.29	789.91***
Household size #	4.71	5.49	17.71***
Women education average	9.92	8.87	−23.62***
Grants	60.83	39.17	626.52***
Remittance	64.47	35.53	0.28***
Improved water	68.48	31.52	265.23***
Improved sanitation	66.54	33.46	159.52***
Clean cooking energy	68.44	31.56	468.93***
Standardized household asset index	−0.08	−1.01	−41.82***

*** Significance at *p* ≤ 0.001, # - household members. Percentages are reported for binary variables and means for continuous variables, with χ^2^ and *t*-tests used to assess statistical significance.

Table 4 shows household characteristics by area of residence. Rural households had higher agricultural participation (78%), larger household sizes (5 members), and received more remittances compared to urban households (54.9%) at *p* ≤ 0.001. Urban households had higher food insecurity (56%), more female-headed households (60.9%), educated women (10.8%), received more grants (52.6%) and had improved access to basic services (>65%). The higher urban food insecurity exceeded rural households contradicting the common assumption that urbanization improves food security.

**Table 4:** Household characteristics by area of residence.

Variable	Rural (%)	Urban (%)	Chi-square/*t*-statistics
Food insecurity experience scale	43.53	56.47	496.49***
Female-headed	39.09	60.91	1,447.85***
Women empowerment index	0.09	0.17	3.82***
Agriculture participation	78.04	21.96	34,065.51 ***
Household size #	5.39	4.35	−64.21***
Women education average	9.23	10.83	95.57***
Grants	47.37	52.63	10,302.06 ***
Remittance	54.91	45.09	2,174.16 ***
Improved water	28.05	71.95	26,315.49 ***
Improved sanitation	31.48	68.52	15,490.86 ***
Clean cooking energy	32.29	67.71	15,883.39 ***
Standardized asset index	−0.52	0.40	102.07***

*** Significance at *p* ≤ 0.001, # - household members. Percentages are reported for binary variables and means for continuous variables, with χ^2^ and *t*-tests used to assess statistical significance.

### 4.3 Logistics regression

Diagnostic checks were conducted for the logit models. Multicollinearity was assessed using variance inflation factors (VIFs), with all values below commonly accepted thresholds, indicating no serious multicollinearity among explanatory variables. For survey-weighted logit regression models estimated using the svy command, traditional likelihood-based goodness-of-fit measures (including pseudo-*R*^2^, AIC/BIC, and ROC-based statistics) are not available due to software limitations with probability weights. Overall model adequacy was assessed using the Wald F test, which is the appropriate global significance test for survey-weighted logistic regression.

The bivariate analysis highlighted strong association between food insecurity and household characteristics suggesting that female headship, agricultural participation, household size and women’s employment, education, access to services and household assets all play important roles. To further examine these relationships while accounting for potential confounding factors and the survey design, we conducted survey-weighted logistic regression analyses (Table 5). All three models (combined, rural, urban) were estimated. Stratified analyses reveal substantial rural–urban heterogeneity, justifying separate interpretation. However, the combined model enables synthesis of common pathways.

**Table 5:** Logistic regression.

Food insecurity experience scale	Combined		Rural		Urban	
	Odds ratio	[95% Conf. interval]	Odds ratio	[95% Conf. interval]	Odds	[95% Conf. interval]
Female head	1.33***	(1.20, 1.48)	0.82*	(0.67, 1.00)	1.66***	(1.46, 1.90)
Agriculture participation	2.01***	(1.78, 2.27)	1.53***	(1.27, 1.83)	1.68***	(1.34, 2.10)
Female head*agriculture participation	0.81***	(0.70, 0.93)	1.26**	(1.01, 1.57)	0.84	(0.62, 1.14)
Women empowerment index	0.76***	(0.67, 0.87)	1.29***	(1.08, 1.54)	0.49***	(0.41, 0.59)
Household size #	1.06***	(1.05, 1.08)	1.06***	(1.04, 1.08)	1.05***	(1.03, 1.07)
Women education average	1.00	(0.99, 1.02)	1.03***	(1.01, 1.05)	0.94***	(0.91, 0.96)
Grants	2.08***	(1.83, 2.37)	1.98***	(1.56, 2.51)	1.75***	(1.48, 2.07)
Remittance	0.97	(0.87, 1.06)	0.98	(0.88, 1.10)	0.86*	(0.72, 1.01)
Improved water	0.94**	(0.84, 0.98)	0.72***	(0.66, 0.79)	1.56***	(1.30, 1.88)
Improved sanitation	0.75***	(0.67, 0.84)	0.69***	(0.61, 0.77)	0.99	(0.76, 1.29)
Clean cook energy	0.88***	(0.80, 0.97)	0.85***	(0.76, 0.95)	0.72***	(0.59, 0.89)
Standardized asset index	0.514***	(0.48, 0.54)	0.57***	(0.53, 0.61)	0.48***	(0.45, 0.52)
Urban	1.88***	(1.71, 2.07)				
Year: 2024	1.68***	(1.57, 1.80)	1.27***	(1.16, 1.40)	2.39***	(2.13, 2.69)
Constant	0.10***	(0.08, 0.12)	0.16***	(0.11, 0.23)	0.17***	(0.11, 0.28)
Mean dependent variable		0.355	0.385		0.318	
Number of observations		17,790	9,892		7,898	
SD dependent variable		0.479	0.487		0.466	
Wald F-test		137.735***	56.772***		89.983***	

*, **,*** Significance at *p* ≤ 0.05, *p* ≤ 0.01 and *p* ≤ 0.001, respectively; # - household members.

The regression results reveal clear gendered patterns in household food insecurity. Several factors were found to be significantly associated with household food insecurity. For the combined model, female headed households had 33% higher odds of experiencing food insecurity compared to male-headed households. Agricultural participation was also positively associated with food insecurity and this was not expected and it contradicts Kabeer’s framework. This indicated that households engaged in agricultural activities tend to experience higher chances of food insecurity. Agricultural household report higher food insecurity likely reflecting limited market access, low productivity, climatic stress and post-harvest losses. The interaction between female headship and agricultural participation implies that this association is attenuated among female-headed household. While engagement in agriculture is generally linked to greater food insecurity, this effect is weaker for female-headed households, reflecting the potential role of subsistence farming or targeted social support programs that benefit women in agriculture.

The women’s empowerment rate reduces food insecurity by 24% lower odds implying that household where more women are employed are less likely to experience food insecurity. Larger household size is associated with higher food insecurity (6% odds). Access to improved water and sanitation, clean cooking energy and higher asset ownership are linked to a lower likelihood of food insecurity. Urban residence had 88% higher odds of food insecurity, indicating that households living in urban areas of the Eastern Cape face greater food access challenges relative to rural households. The year effects were also significant and positive, showing that food insecurity worsened in 2024 relative to 2023.

Disaggregating by place of residence, the rural–urban divide is evident. In rural areas, agricultural participation remains associated with food insecurity, although at lower magnitude than in the combined model. The interaction between female headship and agriculture is positive suggesting that agricultural involvement may exacerbate vulnerability for female-headed households. Conversely, in urban areas, female-headed households remain more food insecure with 66% higher odds, but the interaction effect is insignificant. This was not expected as rural areas are commonly known of experiencing food insecurity and this could be attributed to subsistence agriculture and societal norms. Across models, women’s empowerment emerges as an important factor. While women’s empowerment in rural areas shows a weak or inconsistent relationship, it is strongly protective against food insecurity in urban households. The results also confirms that households with better access to services and higher asset ownership are generally less food insecure. Food-insecure households were more likely to receive grants especially in rural areas. A complementary model estimating the determinants of severe food insecurity produced results that were broadly consistent with those of the main model, reinforcing the robustness of the observed relationships.

### 4.4 Structural equation modelling and mediation analysis

Table 6 shows the structural equation model linking women’s empowerment, resource access, food security and agricultural activities. The model demonstrated good internal consistency with significant paths across most relationships (*p* ≤ 0.001). Standardized coefficients indicated both direct and indirect effects, highlighting empowerment and access to resources as key determinants of livelihood outcomes. Empowerment had the strongest positive influence on access to resources (*β* = 0.441, *p* ≤ 0.001). Urban residence had significant positive effect on access to resources (*β* = 0.223, *p* ≤ 0.001).

**Table 6:** Structural equation model.

Standardized	Direct effects		
	Coefficient	[95% conf. interval]	
Structural		Lower	Upper
RESOURCE ACCESS			
Empowerment index	0.441***	0.428	0.454
Household size	−0.131***	−0.142	−0.12
Urban	0.223***	0.21	0.236
Year 2024	−0.006	−0.019	0.007
Improved water	0.016**	0.002	0.03
Improved sanitation	0.065***	0.053	0.077
Clean cook energy	0.094***	0.082	0.105
Age of household head	0.140***	0.127	0.153
Constant	−1.104***	−1.169	−1.039
AGRICULTURE_PARTICIPATION			
Resource access	−0.037***	−0.053	−0.022
Empowerment index	−0.037***	−0.052	−0.021
Household size	0.131***	0.118	0.145
Urban	−0.489***	−0.504	−0.473
Year 2024	0.011*	−0.002	0.024
Constant	1.055***	1.016	1.095
FIES_MSS			
Resource access	−0.266***	−0.283	−0.25
Agriculture participation	0.113***	0.095	0.132
Empowerment index	−0.043***	−0.06	−0.026
Household size	0.071***	0.056	0.086
Urban	0.099***	0.08	0.119
Year 2024	0.109***	0.095	0.124
Constant	0.215***	0.171	0.259
AGRICULTURE SALES_			
Resource access	0.017*	−0.001	0.035
Agriculture participation	0.203***	0.184	0.221
FIES_MSS	−0.017**	−0.033	−0.002
Empowerment index	0.017	−0.004	0.038
Household size	−0.026***	−0.042	−0.01
Urban	0.051***	0.023	0.078
Year 2024	0.017**	0.001	0.032
Constant	−0.036	−0.089	0.017
var(resource access)	0.606	0.596	0.617
var(e.agriculture participation)	0.68	0.667	0.693
var(e. FIES_MSS)	0.883	0.875	0.892
var(e.agriculture sales)	0.97	0.966	0.975
SRMR	0.022		

*, **,*** Significance at *p* ≤ 0.05, *p* ≤ 0.01 and *p* ≤ 0.001, respectively.

Contrasting results were observed for household size which negatively influenced access to resource (*β* = −0.131, *p* ≤ 0.01). Access to improved water, sanitation and clean cooking services positively influenced resource access (*β* = 0.016–0.094, *p* ≤ 0.05). Agricultural participation was positively associated with household size (*β* = 0.131, *p* ≤ 0.001) and food security (*β* = 0.113, *p* ≤ 0.001), but negatively related to urban residence and empowerment suggesting diversification from subsistence farming among empowered and urban households. Food insecurity negatively affected agricultural sales (*β* = −0.017, *p* ≤ 0.05) emphasizing the role of food security as a prerequisite for market engagement.

Table 7 presents indirect effects of access to basic services on food insecurity. Agricultural participation is positively associated with food insecurity, indicating that households engaged in agriculture tend to be more food insecure. However, improved access to basic services is associated with lower agricultural participation. As a result, access to resources indirectly reduces food insecurity by decreasing reliance on agricultural participation.

**Table 7:** Indirect effects.

Indirect effects	Coefficient	[95% Conf. interval]	
		Lower	Upper
Empowerment index - Resource access - FIES_MSS	−0.058***	−0.062	−0.054
Resource access - Agriculture participation FIES	−0.002***	−0.003	−0.001
Empowerment index - Resource access - Agriculture participation - FIES_MSS	−0.058***	−0.062	−0.054
Resource access - Agriculture participation - agriculture sales	−0.001**	−0.001	0

*, **, *** Significance at *p* ≤ 0.05, *p* ≤ 0.01 and *p* ≤ 0.001, respectively.

**Figure 2:**
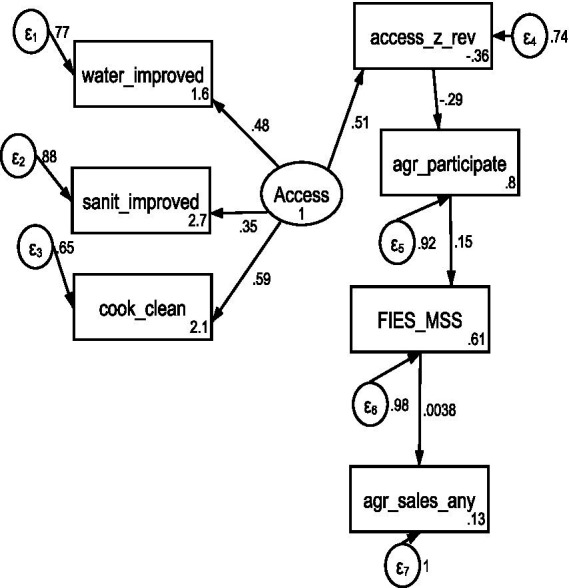
Latent SEM illustrating pathways from access to basic services to agricultural participation and food security.

As a robustness check, we estimated a latent-access SEM using water, sanitation, and clean cooking as indicators (Figure 2). Results are consistent with the main observed-access model. Improved access is associated with lower agricultural participation, while agricultural participation is positively associated with food insecurity. Model fit was moderate (χ^2^(14) = 2647.26, RMSEA = 0.101, CFI = 0.731, TLI = 0.596, SRMR = 0.077), which is expected given the large sample size and the simplified structure of the robustness model.

## 5 Discussion

### 5.1 Gender and household headship

The study investigated the role of women’s empowerment in food security. The analysis of factors influencing household food insecurity using the Food Insecurity Experience Scale (FIES) reveals substantial heterogeneity across rural and urban settings, gendered household structures, and socioeconomic characteristics in the Eastern Cape. Similar findings were reported by Henderson et al. (2024) who reported heterogeneity among rural households highlighting the importance of understating household diversity and variations as they contribute to the food security status. The findings demonstrate that food insecurity is multidimensional, shaped by economic status, gender roles, empowerment, household composition, and access to essential infrastructure and services (Statistics South Africa, 2025). This align with structural and systemic determinants of food insecurity in developing economies (Elum and Emodi, 2025; Haque et al., 2024).

The results for gender of household head and food insecurity indicate that female-headed households in the Eastern Cape are significantly more food insecure than male-headed households. This could be attributed to low prevalence of females in agricultural and income-generating activities (Statistics South Africa, 2025). This aligns with Nkwana and Mazenda (2025) and Akalu and Wang (2023) who postulated that gendered disparity in food security status are possibly linked to patriarchal norms and unequal access to productive resources. As a result, gender-based labor segmentation constrain women’s economic participation (Kocabıçak and Dildar, 2025). The study showed that female headship increases food insecurity in urban areas but reduces it in rural areas. This suggests that while urban female-headed households often depend on volatile informal labor markets and have limited access to productive assets, rural female-headed households may rely on subsistence agriculture and social networks to buffer against hunger and shocks (Egah et al., 2023; Nabweteme et al., 2025). The rural advantage may also reflect strong community-based support systems and household food production that mitigate market dependency. These findings reinforce the importance of context-specific gender-sensitive interventions that consider how women’s vulnerabilities and opportunities differ spatially.

### 5.2 Agricultural participation

The study shows that agricultural participation is positively associated with food insecurity. This was not expected given that agriculture is often viewed as a pathway to food security. This could be attributed to challenges such as limited access to markets, low productivity, climatic stress, and postharvest losses which have been reported to affect smallholder farmers (Kapari et al., 2023; Touch et al., 2024). The results imply that participation in agriculture in either rural or urban areas does not guarantee improved food security, quality and resilience of agricultural engagement is important (Gunapala et al., 2025). Many rural households engage in subsistence production that may not yield sufficient income or food, while urban agriculture may face spatial and regulatory constraints (Touch et al., 2024). Interestingly, food-insecure households may disassociate from agriculture activities, making agricultural participation to be associated with insecurity. Selection effect could have attributed the findings. Therefore, strengthening climate-smart and market-oriented agricultural systems remains vital to ensuring that agricultural participation translates into tangible food security gains.

The study’s findings on the interaction between female household heads and agricultural participation provides additional nuance. The significant coefficient for the combined province indicates that when female-headed households engage in agriculture, food insecurity tends to decline. This suggests that agricultural participation may serve as a resilience strategy for women, enhancing access to food and income (Egah et al., 2023; Sabar et al., 2023; Samputra, 2025). However, the positive and significant coefficient in rural areas indicates that rural female households face unique structural barriers such as limited land rights, inadequate access to credit, and lack of extension services that constrain their productivity (Mkuna and Wale, 2023; Quisumbing et al., 2023; Mugari et al., 2025). Urban female farmers, though fewer in number, may experience smaller yet positive returns from urban agriculture initiatives (Swanepoel et al., 2021). These findings highlight the importance of policies that promote women’s equitable access to agricultural inputs and decision-making processes to maximize the potential of agriculture as a gender-inclusive food security strategy.

### 5.3 Women’s empowerment pathways

The study showed that the role of women’s empowerment is mixed but highly significant. This concurs with Haque et al. (2024) who posited that empowerment reduces food insecurity, highlighting the critical role of agency and decision-making in household welfare. Murugani and Thamaga-Chitja (2019) reported reduced food insecurity vulnerability among households with empowered women in Limpopo. Lufuke et al. (2023) and Jemaneh and Shibeshi (2023) postulated that empowered women are more likely to control household income, influence food purchases, and invest in nutrition and education (Lufuke et al., 2023; Jemaneh and Shibeshi, 2023). Egah et al. (2023) also associated women’s empowerment with improved dietary diversity scores. Results for rural households show that empowerment is positively associated with food insecurity. This could indicate empowerment challenges for women-headed household attributed from male absence as women experience economic strain and increased responsibilities (Ashagidigbi et al., 2022). In urban households, empowerment strongly reduces food insecurity, possibly reflecting the greater economic and educational opportunities available to empowered women in non-agricultural sectors as highlighted by Jemaneh and Shibeshi (2023) and Quisumbing et al. (2023).

Women’s education significantly improved food security in rural areas but reduces it in urban households, likely reflecting differences in labor markets and opportunity structures (Sarker et al., 2024). Rudolph et al. (2024) reported that education enhances food security by improving agricultural productivity and farming strategies. Henderson et al. (2024) reported that higher educational attainment provides better access to agricultural information and employment opportunities which boosts productivity, household income ad food security. Educated women in rural areas may utilize knowledge hubs and networks to improve household welfare. In urban areas, education enables better access to income-generating employment, reducing food insecurity risk (Rudolph et al., 2024; Savari et al., 2020). Haque et al. (2024) also reported that the promotion of formal and informal education is critical in enhancing skills and women’s empowerment ultimately improving household food security.

### 5.4 Household composition and size

The positive relationship between household size and food insecurity across all models aligns with prior findings that larger households face greater challenges in meeting food needs (Mbhenyane and Tambe, 2024). In contrast, Ashagidigbi et al. (2022) observed reduced food insecurity in large households attributing to the involvement of family members in income-generating activities. However, larger families may experience resource dilution effects, especially where income growth is not in line with household expansion. This highlights the need for targeted interventions addressing household dependency ratios and family planning as part of comprehensive food security strategies.

### 5.5 Social protection and services

Social protection mechanisms particularly grants play a crucial role in buffering households against food insecurity. The positive and significant coefficients for grants implied their importance in improving food security. The findings show that food-insecure households are more likely to receive grants. Nengovhela et al. (2024) and Waidler and Devereux (2019) who reported that grants which are part of the South African government efforts toward addressing food security are often inadequate to support larger families due to insufficient funding to cover all household expenses. Chakona and Shackleton (2019) found that households receiving grants experienced higher food insecurity with decreased food access and dietary diversity. This suggested that social grants do not effectively alleviate food insecurity, as recipients often channel their funds to various necessities rather than solely on food (Mbewana and Kaseeram, 2025). Remittances, though significant only in urban households, tend to reduce food insecurity, reflecting the stabilizing role of migrant income. This is in line with Daidone et al. (2020) who reported that social safety nets and remittance flows can act as risk management tools that smooth consumption and support food access during shocks. Zingwe et al. (2023) reported that remittances mitigate food insecurity especially in rural households experiencing multiple shocks.

Access to improved water, sanitation, and clean energy services significantly affects food security outcomes. The results showed that improved sanitation and clean cooking energy reduce food insecurity across most models. This aligns with Mazenda and Nkwana (2025) who reported that energy availability and sanitation improved food security, although sanitation involved several factors such as infrastructure. This highlighted the health–nutrition linkage emphasized in the One Health and Sustainable Development Goal frameworks (Li et al., 2024). However, the urban results for improved water access are unexpectedly positive, potentially reflecting the costs associated with purchasing water in informal settlements or intermittent water supply challenges (Asaki et al., 2024). These results highlight that infrastructure improvements must be coupled with affordability and reliability to achieve meaningful impacts on food security.

### 5.6 Economic asset accumulation

The asset index was one of the strongest predictors of reduced food insecurity. This highlights the importance of economic resilience and capital accumulation. Asset-rich household can better absorb income shocks, invest in productive activities and maintain stable food access (Oluwatayo and Marutha, 2020). This aligns with Majiwa et al. (2025) and Mbewana and Kaseeram (2025) who postulated the linkage of wealth diversification to food security resilience. Furthermore, strengthening household asset bases through access to credit, livestock ownership or productive land remains a cornerstone of sustainable food systems.

### 5.7 Urban–rural dynamics

The positive coefficient for urban residence suggests that urban households are more likely to experience food insecurity, reflecting the vulnerability of low-income urban households that are dependent on food purchases and exposed market volatility. Mkhize and Cele (2025) found that low-income urban dwellers experienced reduced food insecurity owing to the prevalence of indigenous food crop vendors. Nenguda and Scholes (2022) highlighted that urban areas face challenges in social cohesion which erodes social capital and limits the diversity of livelihood strategies for urban households in times of need. Asaki et al. (2024) posited that urban households typically face lower food security than rural ones due to challenges such as higher living costs, reliance on market-based food systems and limited agricultural access. Gunapala et al. (2025), Masuku and Khalema (2024) and Steenkamp et al. (2021) reported that urban populations struggle with affordable, nutritious food access, influenced by high prices and inadequate fresh produce availability. The finding challenges the traditional rural–urban dichotomy and calls for rethinking of food security policies that increasingly consider urban hunger and nutrition transitions. The positive time effect indicated that food insecurity worsened during the period under review. This could be attributed to macroeconomic shocks, inflationary pressures and climate-related stressors affecting food supply chains (Mbajiorgu and Odeku, 2022; Munialo and Mellor, 2024). Although previous research has reported that poverty in the rural areas is higher as compared to urban areas, there is support that urban women, especially those in informal settlements and are female headed experience acute poverty (Statistics South Africa, 2023b). This is supported by Naicker et al. (2015) and Khofi et al. (2024). The result highlights the dynamic nature of food insecurity and the need for continuous monitoring and adaptive policy frameworks.

### 5.8 The SEM pathway findings

The SEM results showed that women play a central role in enabling households to engage in income-generating opportunities. This aligns with Antriyandarti et al. (2024), Khumalo and Zenda (2025), and Sibiya and Oluwatayo (2023) who emphasize empowerment as a catalyst for livelihood diversification and financial inclusion among smallholder farmers. The SEM findings on urban residence suggest that urban households are more integrated into income networks and markets. This is supported by Nenguda and Scholes (2022) and Mkhize and Cele (2025). The negative influence of household size on resource access implied that larger households face greater dependency burdens and resource strain (Adetoro et al., 2023; Bitana et al., 2024). The SEM findings on basic services and resource access implied highlighted the importance of basic service infrastructure in enhancing productivity, health and time allocation for economic activities (Abebe, 2025; Galanakis et al., 2025). The SEM results on agricultural participation highlighted that larger households were more likely to participate in agriculture. This reflects labor availability, while urban households were less engaged due to alternative livelihood opportunities (Hlatshwayo et al., 2023; Wang et al., 2025).

The negative effect of empowerment and access to income on agricultural participation suggest that economically empowered households may diversify out of primary agriculture into non-farm activities (Nontu et al., 2024; Touch et al., 2024). This is consistent with rural transformation, a shift that reflects the growing phenomenon of livelihood diversification as a resilience strategy. The finding that food security enhanced access to resource and agricultural participation confirm that both income diversification and agricultural engagement are essential in reducing food insecurity among households (Silvestri et al., 2015; Mbewana and Kaseeram, 2025). The observed link between food security and empowerment is consistent with the view that decision-making power enhances food utilization and purchasing ability and this aligns with Lufuke et al. (2023). The findings from the study are consistent with literature which argues that empowerment enhances food security through improving access to, control over and utilization of resources (Tesafa et al., 2025; Sarker et al., 2024). This in essence suggests that household resources do play a very important role in mediating the relationship between women’s empowerment and food security. The results imply that empowerment translates into meaningful outcomes through asset accumulation. The association of food security with household size and urban residence implied that urban food access challenges and household size pressures exacerbate vulnerability (Ashagidigbi et al., 2022). The positive coefficient for year 2024 indicated a deterioration in food security conditions, possibly linked to inflation, climate variability or post pandemic stressors in South Africa (Nontu et al., 2024; Galanakis et al., 2025). The results on agricultural sales highlighted that engagement in production is a prerequisite for market participation and urban proximity can facilitate access to markets. The results also show the patterns observed by Touch et al. (2024) and Hlatshwayo et al. (2023) which highlight that market participation among smallholder farmers depends not only on production levels, but also on household food security and empowerment conditions.

## 6 Limitations

It is important to note that while the study identifies significant associations between agricultural participation, female headship, and food insecurity, potential endogeneity between these variables could not be formally tested due to the lack of valid instruments in the available data. The results are therefore interpreted as correlations rather than definitive causal effects. The structural equation modelling analysis does not incorporate survey weights or design-based standard error adjustments, which may lead to underestimated standard errors. Factors such as social capital, health shocks, climate variability, and market access may also influence food insecurity but could not be directly included due to data limitations. The data employed in the study is cross-sectional, which makes it a challenge to infer changes over time. The data is self-reported, however the GHS methodology indicates that the data collection process was more. There are variables which were omitted given the focus of the survey, which if they were included, they could have enhanced the results obtained. The same also applied to the proxies that were employed. Lastly, the study focused on the Eastern Cape, a province with significant challenges. Future studies will consider a comparative analysis between the Eastern Cape and other provinces in South Africa.

## 7 Policy implications and recommendations

The study highlighted the need for integrated livelihood strategies that link social empowerment, infrastructure development and agricultural productivity. Policies that improve access to clean energy, water and sanitation can have significant multiplier effects on both income and food security. Policies that expand grid electrification and/or solar home systems to 90% of rural households by 2030, prioritizing female-headed households. This would reduce food insecurity based on *β* = 0.094 coefficient. Promoting women’s empowerment and inclusive agricultural commercialization can enhance the capacity of smallholder households to engage profitable in agricultural markets while maintaining food security. Interventions must also consider the dual challenges of urbanization and household size which shape both resource availability and market participation. To address vulnerability of female-headed households to food insecurity, enhancement of programs that focus on gender-sensitive social protection, access to productive resources and tailored agricultural support should be prioritized. Empowerment of women through access to land, credit and labor-saving technologies is important for enhancing food security and resilience. It is imperative for support to be provided for agricultural commercialization pathways that move beyond subsistence farming which is associated with higher food insecurity. This will strengthen market access and value chains for smallholder farmers. Participation in agriculture should be encouraged through inclusive access to inputs and markets, supplemented by agroecological practices. Strengthening social protection through expanded grants and improved access to basic services is essential for reducing food security. Specific interventions in urban areas including support for urban agriculture and integration of food security with urban planning are necessary to address the challenges. Promotion of asset accumulation through savings and microfinance can significantly buffer households against food shocks.

Our findings also reveal that agricultural participation in rural areas is influenced by gendered power structures affecting decision-making and control of resources. Despite women’s significant contributions to agricultural work, men typically retain control over land, inputs, and marketing. The success of agricultural engagement on food security hinges on who holds decision-making power. Empowering women is crucial for translating agricultural resources into better food security outcomes. Therefore, policies must focus not only on boosting agricultural production but also on overcoming gender-related barriers in land access and decision-making within households. Improving rural food security requires not only expanding agricultural opportunities but also transforming gendered decision-making structures within households and rural residence.

The differentiated effects of agricultural participation by gender are influenced by rural land governance and customary tenure systems that prefer males. Although reforms exist for gender equality, issues like weak enforcement and entrenched patriarchal norms hinder women’s land control. Insecure tenure diminishes incentives for agricultural investment and limits access to credit and services, negatively impacting food security. Therefore, enhancing rural food security entails not just promoting agricultural involvement but also confronting structural inequalities in land governance. Without secure and enforceable land rights for women, agricultural development strategies risk reinforcing existing gender inequalities and limiting the potential food security gains in rural areas.

## 8 Conclusion

The study investigated the relationships between women’s empowerment, access to resources, agricultural participation and food security in urban and rural households in the Eastern Cape Province, South Africa using structural equation model. Findings show that urban residence and improved sanitation influence access to resources. Larger household sizes limit resource access indicating vulnerability within households. Agriculture participation is affected by urban–rural settings and household size. Rural households face higher risks of food insecurity compared to urban households. The SEM analysis showed that empowerment and improved resource access drive income generation. This in turn enhances food security and agricultural commercialization. Agricultural participation plays a dual role as a direct livelihood activity and as a buffer against food insecurity. Targeted interventions in strengthening empowerment and service delivery in both rural and urban areas are important in cultivating gender quality and food security. This is significant in achieving sustainable development goals and resilient food systems in South Africa.

## Data Availability

Publicly available datasets were analyzed in this study. This data can be found at: Statistics South Africa (2023a).
